# Group I Metabotropic Glutamate Receptor-Mediated Gene Transcription and Implications for Synaptic Plasticity and Diseases

**DOI:** 10.3389/fphar.2012.00189

**Published:** 2012-11-01

**Authors:** Hansen Wang, Min Zhuo

**Affiliations:** ^1^Department of Physiology, Faculty of Medicine, University of TorontoToronto, ON, Canada; ^2^Center for Neuron and Disease, Frontier Institute of Science and Technology, Xi’an Jiaotong UniversityXi’an, China

**Keywords:** group I metabotropic glutamate receptors, gene transcription, CREB, FMRP, signal transduction, synaptic plasticity, fragile X syndrome

## Abstract

Stimulation of group I metabotropic glutamate receptors (mGluRs) initiates a wide variety of signaling pathways. Group I mGluR activation can regulate gene expression at both translational and transcriptional levels, and induces translation or transcription-dependent synaptic plastic changes in neurons. The group I mGluR-mediated translation-dependent neural plasticity has been well reviewed. In this review, we will highlight group I mGluR-induced gene transcription and its role in synaptic plasticity. The signaling pathways (PKA, CaMKs, and MAPKs) which have been shown to link group I mGluRs to gene transcription, the relevant transcription factors (CREB and NF-κB), and target proteins (FMRP and ARC) will be documented. The significance and future direction for characterizing group I mGluR-mediated gene transcription in fragile X syndrome, schizophrenia, drug addiction, and other neurological disorders will also be discussed.

## Introduction

The metabotropic glutamate receptors (mGluRs) belong to family C of G-protein coupled receptors (GPCRs) and are widely distributed throughout the central nervous system (CNS; Kim et al., 2008; Niswender and Conn, 2010; Nicoletti et al., 2011). These receptors are distinguished from family A of GPCRs by the presence of a large extracellular N-terminal domain that contains the endogenous ligand binding site. The mGluRs bind glutamate within a large extracellular domain and transmit signals through the receptor protein to intracellular molecular partners, and provide a mechanism by which glutamate modulates cell excitability and synaptic transmission via second messenger signaling pathways (Kim et al., 2008; Gladding et al., 2009). Eight mGluR subtypes have been identified and are subclassified into three groups based on sequence homology, G-protein coupling, and ligand selectivity. Group I mGluRs (mGluR1 and mGluR5) are predominantly coupled to the activation of phospholipase C (PLC) *via* Gαq/11, whereas group II (mGluR2 and mGluR3) and group III (mGluR4, mGluR6, mGluR7, and mGluR8) mGluRs negatively regulate adenylyl cyclase *via* Gαi (Niswender and Conn, 2010; Nicoletti et al., 2011). These mGluR subtypes are differentially localized in presynaptic and postsynaptic regions of neurons, as well as in glial cells (Kim et al., 2008; Gladding et al., 2009; Ribeiro et al., 2010).

The mGluRs can modulate diverse neuronal responses and their downstream targets exist not only in the membrane but also in the cytoplasm and nucleus (Heinke and Sandkuhler, 2005; Gerber et al., 2007; Gladding et al., 2009). Group I mGluRs have been shown to regulate gene expression at both translational and transcriptional levels (Antar et al., 2004; Hou et al., 2006; Gerber et al., 2007; Gladding et al., 2009). Previous studies have provided insights into how mGluRs initiate signal transduction and details about the signaling pathways downstream of these receptors. The roles of group I mGluRs in protein synthesis have been well documented (Bear et al., 2004; Garber et al., 2006; Hou et al., 2006; Waung et al., 2008; Waung and Huber, 2009). Here, we will focus on group I mGluR-mediated gene transcription and its implications in synaptic plasticity. We will discuss how group I mGluRs induce gene transcription and the significance of group I mGluR-mediated gene transcription in neurological conditions.

## Group I mGluR-Mediated Gene Transcription

### Group I mGluR signaling pathways

**Figure 1:**
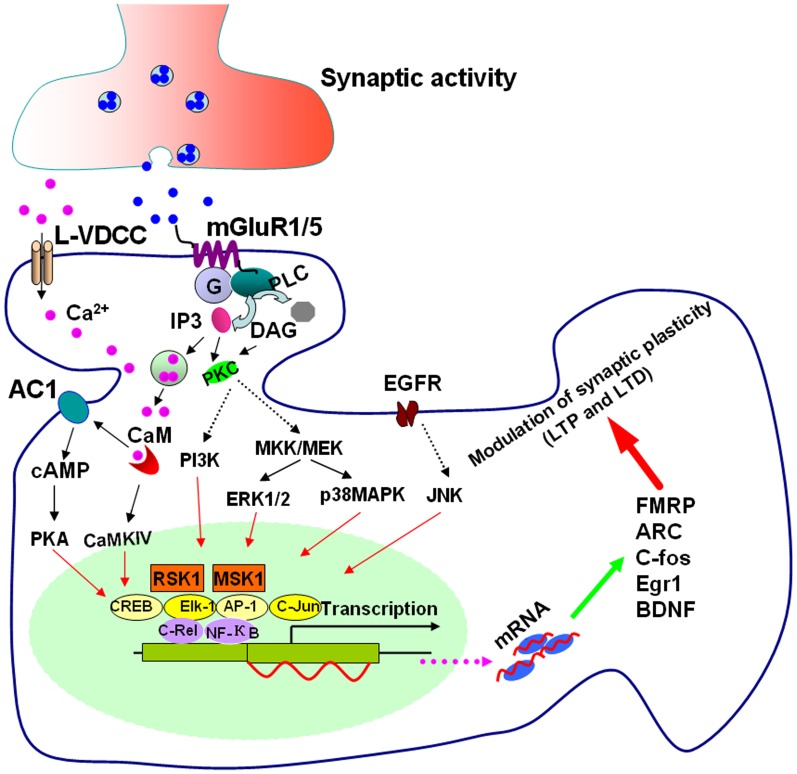
**Signaling pathways for group I mGluR-mediated gene transcription**. Stimulating group I mGluRs triggers Ca^2+^ release from intracellular calcium stores by IP3 and Ca^2+^ influx from L-VDCCs through membrane depolarization. Postsynaptic increases of Ca^2+^ lead to activation of Ca^2+^-calmodulin (CaM) dependent pathways. Among them, AC1 and CaMKIV are activated. AC1 activation leads to the generation of cAMP and cAMP then activates PKA. PKA and CaMKIV phosphorylate CREB. ERK1/2 and p38 MAPK are stimulated by G-protein release after mGluR1/5 activation through the mitogen-activated protein kinase kinases MKK/MEK. JNK can be stimulated by transactivation of EGFR by group I mGluRs. PI3K is also initiated by group I mGluR activation. Stimulation of ERK1/2, p38 MAPK, JNK, and PI3K leads to activation of the transcription factors Elk-1, CREB, activator protein-1 (AP-1), c-Jun, and other NF-κB members such as c-Rel through RSK1 and mitogen and MSK1. The upregulation of the targets such as FMRP, ARC, c-fos, Egr1, and BDNF by these transcriptional factors, could contribute to the modulation of synaptic plasticity in the forms of LTP and LTD.

Group I mGluRs are primarily coupled to the activation of Gα_q/11_ proteins which stimulate PLCβ, resulting in the cleavage of phosphatidylinositol-4,5-bisphosphate with the ensuing formation of the intracellular second messengers, inositol-1,4,5-trisphosphate (IP3), and diacylglycerol (DAG; Gladding et al., 2009; Niswender and Conn, 2010; Nicoletti et al., 2011). Additionally, group I mGluR activation facilitates L-type voltage dependent Ca^2+^ channels (L-VDCCs) and induces Ca^2+^ influx through L-VDCCs (Chavis et al., 1996; Mao and Wang, 2003b). The IP3 binding to its receptor leads to the release of Ca^2+^ from intracellular stores; both Ca^2+^ and DAG activate protein kinase C (PKC); PKC has been found to activate phospholipase D (PLD), phospholipase A2 (PLA2), as well as to modulate a variety of ion channels (Niswender and Conn, 2010; Nicoletti et al., 2011). It is known that group I mGluRs can trigger additional signaling pathways downstream of Gα_q/11_ as well as pathways stemming from Gαs and Gαi/o, and other molecules independent of G-protein (Gerber et al., 2007; Kim et al., 2008; Niswender and Conn, 2010). Depending on the neuronal populations, stimulating group I mGluRs can activate a wide range of protein kinase pathways, including cAMP dependent protein kinase (PKA), Ca^2+^ calmodulin dependent protein kinases (CaMKs), mitogen-activated protein kinases (MAPKs), phosphoinositide 3-kinase (PI3K), mammalian target of rapamycin (mTOR), p70 S6 kinase, casein kinase 1, and cyclin-dependent protein kinase 5 (Liu et al., 2002; Harris et al., 2004; Hou et al., 2006; Mao et al., 2008; Gladding et al., 2009; Niswender and Conn, 2010; Ribeiro et al., 2010; Nicoletti et al., 2011). These signaling pathways are believed to be particularly important for the regulation of synaptic plasticity by group I mGluRs. The PKA, CaMKs, MAPKs, and PI3K pathways have been shown to link group I mGluRs to transcriptional changes in the nucleus (O’Riordan et al., 2006; Gerber et al., 2007; Gladding et al., 2009; see Figure 1 for the model).

#### PKA

The cAMP signaling pathway contributes to the activity-dependent synaptic plasticity in the anterior cingulate cortex (ACC; Wei et al., 2002b, 2006; Liauw et al., 2005; Zhuo, 2008). Previous studies have shown that stimulating Group I mGluRs potentiates cAMP accumulation in cultured striatal neurons, striatum, and cerebral cortex (Cartmell et al., 1997, 1998; Schaffhauser et al., 1997). We found that stimulating group I mGluRs with their agonist (RS)-3, 5-Dihydroxyphenylglycine [(RS)-3, 5-DHPG], increases the cAMP levels in cingulate cortical neurons (Wang et al., 2008a). Both the intracellular calcium stores and external calcium influx through L-VDCCs are required for cAMP production following stimulation of Group I mGluRs. PKA is activated by Group I mGluRs in ACC neurons (Wang et al., 2008a). Among the cAMP signaling molecules, adenylyl cyclase 1 (AC1) and AC8, are the two major Ca^2+^/calmodulin stimulated AC isoforms (Sunahara and Taussig, 2002; Wang and Storm, 2003; Cooper and Crossthwaite, 2006; Wang et al., 2007a, 2011). We found that AC1, but not AC8, plays a critical role in PKA signaling pathway during group I mGluR activation in ACC neurons (Wang et al., 2008a).

Similarly, group I mGluR agonists have been found to activate PKA in other brain region (Bandrowski et al., 2001). The PKA signaling pathway is critically involved in the effect of group I mGluRs on the state of phosphorylation of GluA1 subunit of the AMPA glutamate receptors (Dell’anno et al., 2012). In amygdala, mGluR5 has been shown to activate PKA signaling pathway through IP3 and reactive oxygen species (ROS; Li et al., 2011).

#### CaMKs

CaMKIV is a key effector in neuronal Ca^2+^ signaling and functions as a transcriptional activator (Ho et al., 2000; Hook and Means, 2001; Wei et al., 2002a; Colomer and Means, 2007; Wayman et al., 2008, 2011). It is expressed in both nuclei and cytosol of neurons in different brain regions, including cortex, striatum, hippocampus, amygdale, and cerebellum (Colomer and Means, 2007; Wayman et al., 2008, 2011). CaMKIV has been implicated in many aspects of neuronal Ca^2+^ signaling, including gene expression in response to excitatory neurotransmission (Colomer and Means, 2007; Wayman et al., 2011). We found that stimulating group I mGluRs activates CaMKIV in the ACC neurons (Wang et al., 2009a). Both Ca^2+^ release from intracellular stores and Ca^2+^ influx through L-VDCCs are involved in the activation of CaMKIV by group I mGluRs in ACC neurons (Wang et al., 2009a). Our study thus suggests that CaMKIV may act as a downstream effector for Group I mGluRs in ACC neurons. In cultured striatal neurons, activation of both cell Surface and intracellular mGluR5 increases phosphorylation of CaMKIV (Jong et al., 2009; Kumar et al., 2012).

Another member of the CaMKs family, CaMKII, is also well known for its roles in gene transcription, synaptic plasticity, learning, and memory (Wayman et al., 2008, 2011; Mockett et al., 2011). DHPG can cause a transient increase in CaMKII phosphorylation in synaptoneurosomes prepared from whole hippocampus and in CA1 slices (Mockett et al., 2011). In striatal neurons, activation of intracellular mGluR5 increases phosphorylated CaMKII (Jong et al., 2009; Kumar et al., 2012). These findings may implicate CaMKII in group I mGluR-mediated gene transcription.

#### MAPKs

Mitogen-activated protein kinases are a family of serine/threonine protein kinases, including extracellular signal regulated kinases (ERKs), p38 MAPKs, and c-Jun N-terminal kinase (JNK; Davis and Laroche, 2006; Wang et al., 2007b). It has been demonstrated that stimulation of Group I mGluRs activates all these three subclasses. The group I mGluRs actively regulate the phosphorylation of MAPKs (Niswender and Conn, 2010; Ribeiro et al., 2010; Nicoletti et al., 2011). DHPG can induce the phosphorylation of ERK1/2 in striatal neurons *in vivo* and in cultures (Mao et al., 2005, 2008). This effect was mediated partially by the conventional group I mGluR signaling pathway since the PLC inhibitor and Ca^2+^ depleting agents or Ca^2+^ chelators reduced DHPG-induced phosphorylation of ERK1/2 (Mao et al., 2005). ERK signaling has been found to be involved in chemical mGluR1-mediated late long-term potentiation (LTP) at excitatory synapses onto hippocampal interneurons in oriens-alveus (OA-INs), suggesting stimulating mGluR1 may activate ERK (Ran et al., 2009, 2012). ERK activation may be triggered by mGluR5 activation by a cascade involving the repressor activator protein 1 (Rap1) and mitogen-activated protein/ERK kinase (MEK; Morozov et al., 2003). In amygdala, ERK may be activated by mGluR5 through IP3 and ROS (Li et al., 2011). Stimulating group I mGluR activates ERK in hippocampus (Hou et al., 2006; O’Riordan et al., 2006). The activation of ERK2 requires PI3K and PKC; Both PI3K and PKC can cause ERK2 activation through Ras signaling pathway and its downstream target MEK, the kinase responsible for activation of ERK (O’Riordan et al., 2006). Group I mGluR-mediated signaling also activates p38 MAPK, whereas DHPG-induced activation of p38 MAPK is independent of PI3K and PKC (Huang et al., 2004; O’Riordan et al., 2006). It has been shown that stimulating mGluR5 can activate p38 MAPK through Rap1 and MAPK kinase 3/6 (MKK3/6; Huang et al., 2004).

In cultured striatal neurons, activation of both cell Surface and intracellular mGluR5 increases phosphorylation of JNK (Kumar et al., 2012). Stimulating group I mGluRs with DHPG induces the phosphorylation of JNK through a signaling mechanism involving mGluR5-associated transactivation of the epidermal growth factor receptor (EGFR), whereas the conventional signaling pathways (IP3 mediated Ca^2+^ release and PKC) are not involved in this process (Yang et al., 2006). Interestingly, the scaffolding protein Homer1b/c may couple mGluR5 to ERK signaling cascade in a Ca^2+^ independent manner (Mao et al., 2005). In addition, another scaffolding protein, caveolin-1, an adaptor protein that associates with lipid rafts and the main protein of caveolae, interacts with group I mGluRs and regulates mGluR-dependent phosphorylation/activation of MAPKs (Francesconi et al., 2009). The studies summarized here demonstrate that group I mGluRs are tightly linked to MAPK signaling pathway.

The group I mGluRs are not only involved in gene transcription, but also contribute to other aspects of gene expression at the post-transcriptional and translational levels (Gerber et al., 2007; Gladding et al., 2009; Nicoletti et al., 2011). It could be argued that PKA, CaMKs, and MAPKs are involved in molecular/cellular processes other than gene transcription. However, our previous studies have provided clear evidence that PKA and CaMKIV are involved in group I mGluR-mediated gene transcription (Wang et al., 2008a, 2009a). The MAPK and PI3K signaling pathways have also been shown to modulate gene transcription through transcription factors by other studies (Gerber et al., 2007; Gass and Olive, 2008; Gladding et al., 2009). The fact that the same signaling pathways could be involved in different molecular/cellular processes further reveals the complexity of group I mGluR-mediated signaling network and the diverse functions of mGluRs.

### Transcription factors

#### CREB

The cyclic AMP responsive element binding protein (CREB) is a transcription factor that plays important roles in synaptic plasticity (Kornhauser et al., 2002; Lonze and Ginty, 2002; Josselyn and Nguyen, 2005; Zhuo, 2005; Suzuki et al., 2011; Kandel, 2012). The activity of CREB is regulated by its phosphorylation. Phosphorylated CREB binds to the CRE site within the gene and activates the gene transcription (Kornhauser et al., 2002; Lonze and Ginty, 2002; Kandel, 2012). We have demonstrated that group I mGluR activation induce the phosphorylation of CREB in ACC neurons (Wang et al., 2008a). AC1 is involved in this process through PKA. CaMKIV is another key molecule that phosphorylates CREB during group I mGluR activation in ACC neurons (Wang et al., 2008a, 2009a).

In striatal neurons, mGluR5 activation induces CREB phosphorylation (Choe and Wang, 2002; Mao and Wang, 2003b; Mao et al., 2008). In hippocampal interneurons, induction of mGluR1-mediated late LTP has been found to stimulate CREB phosphorylation via ERK signaling (Ran et al., 2012), suggesting group I mGluR-induced CREB activation can occur in different brain areas.

#### NF-κB

The nuclear factor κB (NF-κB) family of dimeric transcription factors plays a role in the induction of synaptic plasticity and formation of long-term memory (Sheridan et al., 2007; Ahn et al., 2008; Bracchi-Ricard et al., 2008; Boersma et al., 2011). NF-κB subcellular distribution, DNA binding activity, and transcription are regulated by various forms of synaptic activity (Memet, 2006; O’Mahony et al., 2006; Romano et al., 2006). Moreover, regulation of gene expression by group I mGluRs in cortical neurons requires the activation of NF-κB factor c-Rel (Pizzi et al., 2005), suggesting that NF-κB could be involved in the induction of transcription-dependent forms of synaptic plasticity in the nervous system, including long-term depression (LTD; Pizzi et al., 2005). NF-κB is activated by group I mGluRs in hippocampus (O’Riordan et al., 2006). The DNA binding activity of NF-κB family members, such as p50, p65, and c-Rel, is increased in response to group I mGluR signaling (O’Riordan et al., 2006). The mGluR5, but not mGluR1, is involved in this activation. The PI3K, PKC, MKK, and p38 MAPK signaling pathways are necessary for group I mGluR-induced activation of NF-κB (O’Riordan et al., 2006). In glial cells, glutamate activates NF-κB through mGluR5. The regulation of NF-κB requires Ca^2+^ and cross coupled signaling with EGFR (Sitcheran et al., 2008). These findings reveal the diversity of signaling pathways that modulate NF-κB activity and indicate the importance of cross talks among different classes of receptors to regulate gene expression.

#### Other transcription factors

In striatal neurons, mGluR5-mediated JNK activation can induce the phosphorylation of c-Jun. The phosphorylated c-Jun then leads to activation of the transcription factor activator protein-1 (AP-1) to facilitate target gene expression (Yang et al., 2006). Activation of mGluR5 induced a rapid and transient phosphorylation of the transcription regulator Elk-1 in cultured striatal neurons. Elk-1 phosphorylation was mediated through selective activation of mGluR5 regulated PLC and IP3-sensitive Ca^2+^ release (Mao and Wang, 2003a). ERK signaling targets multiple transcription control pathways in addition to CREB. ERK targets both Elk-1 and CREB in LTP-dependent transcription in dentate gyrus (Davis and Laroche, 2006; Gladding et al., 2009). Stimulation of MAPKs and PI3K leads to activation of the transcription factors Elk-1, CREB, AP-1, c-Jun, and NF-κB through p90 ribosomal S6 kinase 1 (RSK1) and mitogen and stress-activated protein kinase 1(MSK1; Arthur et al., 2004; Hauge and Frodin, 2006; Gladding et al., 2009; Pearce et al., 2010).

### Target gene expression

#### FMRP

Fragile X mental retardation protein (FMRP), an mRNA binding protein, regulates local protein synthesis at synapses and is involved in activity-dependent synaptic plasticity (Bassell and Warren, 2008; Wang et al., 2008b, 2010, 2012b; Bhakar et al., 2012). The lack of FMRP due to mutations in its encoding gene *FMR1* can cause fragile X syndrome, the most common form of inherited mental retardation and autism spectrum disorders (Bear et al., 2004; Garber et al., 2006; Bassell and Warren, 2008; Rooms and Kooy, 2011; Bhakar et al., 2012; Wang et al., 2012b). Previous studies showed that the group I mGluR agonist DHPG induces the increase of FMRP in a translation-dependent manner, and the induction depends on the activation of mGluR5, rather than mGluR1 in hippocampus (Antar et al., 2004; Hou et al., 2006; Bhakar et al., 2012). We found that upregulation of FMRP by group I mGluRs occurs at the transcriptional level in cingulate cortex (Wang et al., 2008a). In addition, the upregulation of FMRP depends on the activation of both mGluR1 and mGluR5 in ACC neurons (Wang et al., 2008a). The differences in our findings from others’ could be explained by the different composition of neuronal types between hippocampus and cingulate cortex. Alternatively, the differences might reflect the possible functional diversity of group I mGluRs in different brain areas. We further demonstrated that CREB is involved in the upregulation of FMRP by group I mGluRs in ACC neurons (Wang et al., 2008a, 2009a). The regulation of FMRP by group I mGluRs requires AC1 and CaMKIV; AC1 and CAMKIV contribute to FMRP upregulation through CREB activation (Wang et al., 2008a, 2009a). By using the transgenic mice overexpressing dominant active CREB (Y134F) mutant which displays a higher affinity with cAMP dependent kinase (PKA) compared to wild-type CREB (Suzuki et al., 2011), we recently demonstrated that upregulation of FMRP by stimulating group I mGluRs was further enhanced in ACC neurons from these transgenic mice, providing further evidence that FMRP upregulation by group I mGluRs involves CREB (Wang et al., 2012a). Our studies thus indicate that FMRP can be transcriptionally regulated by group I mGluRs, and CREB is the key transcription factor in group I mGluR-induced FMRP upregulation in cingulate cortex (Figure 1).

#### ARC

Activity-regulated cytoskeleton-associated protein (*Arc*), also known as activity-regulated gene 3.1 (*Arg3.1*), is an immediate early gene that links gene expression changes with localized synaptic changes (Bramham et al., 2008; Alberi et al., 2011; Beique et al., 2011; Korb and Finkbeiner, 2011; Shepherd and Bear, 2011; Okuno et al., 2012). The expression of *Arc* is tightly coupled to synaptic activities (Alberi et al., 2011; Beique et al., 2011; Korb and Finkbeiner, 2011; Shepherd and Bear, 2011). Previous studies have shown the functional relevance of *Arc* translation in mGluR-dependent LTD in hippocampus (Alberi et al., 2011; Beique et al., 2011; Korb and Finkbeiner, 2011; Shepherd and Bear, 2011). The group I mGluR agonist DHPG can increase dendritic ARC protein levels in a translation-dependent manner (Park et al., 2008; Waung et al., 2008). An increase in Arc transcription was also observed 20 min following DHPG application (Park et al., 2008), suggesting that following rapid translation of Arc, Arc can be upregulated at the transcriptional level. In hippocampal neurons cultured within microfluidic chambers, DHPG applied to synaptic regions is sufficient to signal to the nucleus and increase Arc transcription. The increase in *Arc* mRNA was from new transcription since it can be blocked by the transcription inhibitor Actinomycin D. Interestingly, these newly transcribed *Arc* mRNAs caused by local group I mGluR activation can be delivered to the region of synaptic stimulation (Taylor et al., 2010). This study indicates that group I mGluR activation can prompt a synapse-to-nucleus-to-synapse signaling pathway. In cultured cortical neurons, it has also been demonstrated that activation of group I mGluRs by DHPG can induce transcription-dependent expression of ARC. The DHPG-induced ARC upregulation requires CaMK, PLC, and ERK1/2 activity. Among these signaling molecules, ERK1/2 plays the central role in group I mGluR-mediated *Arc* transcription (Wang et al., 2009b).

#### Other immediate early genes

The transcription of the immediate early genes c*-fos* and *egr1* is dependent on CREB activity (Impey and Goodman, 2001; Kornhauser et al., 2002; Lonze and Ginty, 2002; Josselyn and Nguyen, 2005; Zhuo, 2005; Kandel, 2012). The mGluR5 stimulation upregulates c-fos and egr1 in striatal neurons (Mao and Wang, 2003b,c; Jong et al., 2009). The induction of immediate early genes kinetically corresponds to Elk-1 phosphorylation (Jong et al., 2009) and can be attenuated by selectively knockdown of Elk-1 (Choe and Wang, 2001; Mao and Wang, 2003a).

## Group I mGluR-Mediated Gene Transcription and Synaptic Plasticity

Activation of mGluRs results in diverse actions on neuronal excitability and synaptic transmission by modulation of a variety of ion channels and other regulatory or signaling proteins (Niswender and Conn, 2010; Ribeiro et al., 2010; Nicoletti et al., 2011). Group I mGluRs are usually localized postsynaptically, and their activation can lead to cell depolarization and increases in neuronal excitability. Group I mGluRs play important roles in induction or maintenance of long lasting forms of synaptic plasticity, including LTD and LTP of transmission at glutamatergic synapses (Huber et al., 2000; Huemmeke et al., 2002; Bear et al., 2004; Heinke and Sandkuhler, 2005; Nosyreva and Huber, 2006; Anwyl, 2009; Gladding et al., 2009; Nicoletti et al., 2011).

The mGluR-LTD involves not only the dendritic synthesis of target proteins such as FMRP, ARC, postsynaptic density (PSD) 95, microtubule-associated protein 1B (MAP1B), and striatal-enriched tyrosine phosphatase (STEP), but also the modulation of transcription factors such as NF-κB, Elk-1, and CREB (Gladding et al., 2009). NF-κB is a transcription factor that is activated in mGluR-LTD (O’Riordan et al., 2006). The NF-κB family member c-Rel has been shown to be necessary for long-term maintenance of hippocampal mGluR-LTD (O’Riordan et al., 2006; Ahn et al., 2008). This is inconsistent with previous studies which have shown that mGluR-LTD involves translational rather than transcriptional regulation. The explanation could be that synaptic changes were monitored by previous studies at an early phase (<90 min) rather than at a late phase (2∼3 h) of LTD (Huber et al., 2000; O’Riordan et al., 2006). The inconsistency may also indicate that the early phase of mGluR-LTD depends on dendritic translation of pre-existing mRNA, whereas the late phase mGluR-LTD requires new transcription. NF-κB can be synaptically localized and upon synaptic activation is rapidly trafficked from the synapses to the nucleus (Memet, 2006; Romano et al., 2006). In the stabilization of mGluR-LTD, NF-κB may act as a signal messenger to facilitate the expression of specific genes which are required for maintenance of synaptic activity (O’Riordan et al., 2006).

The long-term synaptic plasticity in hippocampal inhibitory interneurons plays important roles in learning and memory (Bartos et al., 2007; Kullmann and Lamsa, 2007). Activation of mGluR1 at excitatory synapses onto hippocampal interneurons in oriens-alveus can induce a transcription and translation-dependent form of LTP which persists for at least 24 h (Ran et al., 2009). CREB knockdown impaired this mGluR1-mediated chemical late LTP, whereas CREB overexpression facilitated the induction, indicating that CREB-dependent transcription is a necessary permissive switch for eliciting persistent presynaptic and postsynaptic changes at excitatory synapses in inhibitory local circuits (Ran et al., 2012). The CREB-dependent transcription may eventually enhance transmitter release and increases channel conductance and number of functional postsynaptic receptors during maintenance of interneuron persistent synaptic plasticity.

## Implications in Neurological Disorders

A large body of studies have implicated group I mGluRs in the pathogenesis of multiple neurological disorders, including fragile X syndrome, schizophrenia, drug addiction, chronic pain, neurodegenerative diseases, and other neurological disorders (Gravius et al., 2010; Ribeiro et al., 2011; Bellone and Mameli, 2012; Bhakar et al., 2012; Brown et al., 2012; Chiechio and Nicoletti, 2012; Duncan and Lawrence, 2012; Wang et al., 2012b). Targeting individual mGluR subtypes has also shown promising outcomes for treatment of some neurological and psychiatric disorders (Spooren et al., 2010; Gregory et al., 2011; Gross et al., 2012). However, group I mGluR signaling alterations in many of these disorders still need to be further investigated.

The mGluR-mediated LTD is, under certain circumstances, dependent on protein synthesis occurring as a result of local mRNA translation. In the absence of FMRP, LTD associated proteins are constitutively and highly expressed, and this makes a selective amplification of mGluR5 mediated LTD in the hippocampus of *Fmr1* knockout (KO) mice, an animal model of fragile X syndrome (Huber et al., 2000; Bear et al., 2004; Waung and Huber, 2009; Krueger et al., 2011). The transcriptional regulation of gene expression is also required for long-term maintenance of hippocampal LTD and induction of mGluR-mediated late LTP (Memet, 2006; Ran et al., 2012). However, little is known about the gene transcription-dependent mGluR-mediated LTP or LTD in fragile X syndrome. Our previous studies have shown the deficits in LTP in both cingulate cortex and hippocampus of *Fmr1* KO mice (Zhao et al., 2005; Shang et al., 2009). The glycine induced LTP depends on group I mGluRs and is deficient in hippocampus of *Fmr1* KO mice, whereas the function of group I mGluRs is not affected in these mice (Wang et al., 2008a; Shang et al., 2009). Since we have demonstrated that stimulating group I mGluRs upregulates FMRP through CREB (Wang et al., 2008a, 2009a, 2012a), it is likely that the downstream effect of this signaling pathway (group I mGluRs-CREB-FMRP) will be affected when FMRP is absent and thus cause alterations in synaptic responses in fragile X syndrome.

Besides FMRP, many other effectors in signaling pathway of group I mGluR-mediated gene transcription, such as CREB, NF-κB, ARC, and c-fos, have been shown to be involved in chronic pain, drug addiction, neurodegenerative diseases, and psychiatric disorders (Zhuo, 2005; Memet, 2006; Korb and Finkbeiner, 2011; Kandel, 2012). Despite the fact that the functions of these downstream effectors of mGluRs in learning, memory, and synaptic plasticity have been well documented, future research is definitely needed to characterize group I mGluR-mediated gene transcription in neurological and psychiatric disorders.

## Conclusion

Our understanding of mGluRs has greatly advanced in the past decade. Previous studies regarding the function of group I mGluRs have contributed to the knowledge about group I mGluRs in modulation of neural plasticity and therapeutic perspectives of these receptors in neurological disorders. The group I mGluRs are not only involved in protein synthesis in cytoplasm, but also in gene transcription in nucleus. Although group I mGluR signaling pathways are becoming characterized, data on how the mGluR signaling pathways can affect disease development remains limited for most neurological diseases. An important focus of future studies will be the linking of molecular data on group I mGluRs with specific neural and behavioral functions in both physiological and neurological conditions. We hope that the future studies will continue to enhance our understanding of the diverse neuronal mechanisms of mGluRs and will open up new avenues for treatments of neurological and psychiatric disorders.

## Conflict of Interest Statement

The authors declare that the research was conducted in the absence of any commercial or financial relationships that could be construed as a potential conflict of interest.
